# Janus Kinase inhibitors in the treatment of large vessel vasculitis: a systematic review and meta-analysis

**DOI:** 10.1515/med-2026-1387

**Published:** 2026-03-06

**Authors:** Yang Bai, Zhe Wang, Chunling Zhang

**Affiliations:** Department of Rheumatology, Central Hospital Affiliated to Shandong First Medical University, Shandong First Medical University, Jinan, Shandong, China; Department of Traditional Chinese Medicine, Jinan People’s Hospital, Jinan, Shandong, China

**Keywords:** vessel vasculitis, Janus Kinase inhibitors, remission, relapse, adverse event

## Abstract

**Objectives:**

This meta-analysis evaluates Janus Kinase (JAK) inhibitors’ efficacy and safety in large vessel vasculitis (LVV), encompassing Giant Cell Arteritis (GCA) and Takayasu’s Arteritis (TAK).

**Methods:**

Registered on PROSPERO (CRD42024575313), four databases were systematically searched for randomized controlled trials (RCTs) and observational studies. Outcomes included remission/relapse rates and adverse events, assessed with sensitivity/publication bias analyses.

**Results:**

Five studies (107 patients: 65 GCA, 42 TAK) showed pooled 12-month remission rates of 80.1 % (95 % CI: 68.3–92.0), with subgroup rates of 87.3 % (GCA) and 69.6 % (TAK). Relapse rates were 11.3 % overall (2.5 % GCA vs. 22.9 % TAK). Adverse event-related discontinuation was 4.4 %, with subgroup rates of 4.0 % (GCA) and 4.2 % (TAK).

**Conclusions:**

JAK inhibitors demonstrate favorable efficacy and safety in LVV.

## Introduction

Large Vessel Vasculitis (LVV) is a complex autoimmune disease mainly involving the aorta and its branches, which can lead to vascular wall inflammation, stenosis, occlusion and even aneurysm formation, seriously affecting the quality of life and prognosis of patients [1]. These diseases include Giant Cell Arteritis (GCA) and Takayasu’s arteritis (TAK) [2]. Among them, GCA is more common in the European population over 50 years old, mainly involving the temporal artery, while TAK is a systemic granulomatous vasculitis, mainly affecting young women (<40 years old) in Asian countries. According to the 2021 guidelines developed by the American College of Rheumatology/Vasculitis Foundation, glucocorticoid (GC) and immunosuppressants are recommended as initial treatment for microvasculitis [3]. Although GCs and immunosuppressants are able to control the disease to a certain extent, there is a significant risk of adverse reactions and recurrence with long-term use [4], 5]. Therefore, it is particularly important to explore new therapeutic strategies.

In recent years, Janus Kinase (JAK)/Signal Transducer and Activator of Transcription (STAT) signaling pathway as a key way of regulating immune response and inflammatory response, and gradually become a new target for the treatment of autoimmune diseases. The JAK family includes JAK1, JAK2, JAK3 and TYK2 members, which are widely expressed in a variety of immune cells and participate in the regulation of signal transduction of a variety of cytokines. As a new class of small-molecule targeted drugs, JAK inhibitors specifically block the activity of JAK, inhibit the phosphorylation and nuclear translocation of downstream STAT proteins, and thus block the cytokine mediated inflammatory response and immune response. Many immune studies have shown that JAK/STAT signal transduction pathways play an important role in the progression of GCA and TAK diseases [6], [7], [8], [9], [10]. JAK inhibitors can improve symptoms, reduce inflammatory markers, delay disease progression and reduce hormone dependence in patients with LVV [10], 11].

Due to the differences in study design, patient population, therapeutic dose and follow-up time, the comprehensive evaluation of JAK inhibitors in the treatment of LVV is still insufficient. This meta-analysis systematically reviewed and comprehensively analyzed published clinical trial data on JAK inhibitors in the treatment of LVV, evaluated the efficacy and safety of JAK inhibitors, and provided more comprehensive and objective evidence support for clinical decision-making.

## Methods

### Study design and search strategy

This systematic review was conducted in accordance with the Preferred Reporting Items for Systematic Reviews and Meta-Analyses (PRISMA) reporting guidelines. This study was registered with PROSPERO (CRD42024575313). The PubMed, Embase, EBSCO, Scope, Cochrane Library, and Web of Science databases were systematically searched for relevant studies, the latest date of search was August 21, 2024. The following MeSH and free words were used in the searches: “Janus Kinase inhibitors OR JAK inhibitors OR JAKi OR Tofacitinib OR Baricitinib OR Ruxolitinib OR Upadacitinib OR delgocitinib OR filgotinib” AND “arteritis OR vasculitis OR takayasu OR giant cell arteritis”. The language was confined to English.

### Selection criteria

The inclusion criteria were as follows: (1) patients were diagnosed with LVV; (2) patients were treated with JAK inhibitors, either with single-agent therapy or in combination with other treatments; (3) randomized controlled trials (RCTs) or retrospective analysis; and (4) patients were reported the response rate, relapse rate, and adverse events.

The exclusion criteria were as follows: (1) letters, editorials, guidelines, consensus, animal experiments, cell research, reviews, meta-analyses, duplicates; (2) studies with patient number less than 10; and (3) full text or valid data were not available for extraction.

### Data extraction and quality assessment

The title and abstracts of articles were screened by two researchers independently, and required data from all included studies were also independently extracted by two investigators. Disagreements during cross-checking were resolved by a discussion with a third person, and the quality assessment of the studies was performed afterward. The extracted characteristics were summarized as follows: authors, publication year, nation, sample size, median age, median follow-up, types of JAK inhibitors and LVV, previous biologic drugs, and reported endpoints. Indexes for clinical and safety outcomes included remission, relapse, and adverse events. The Newcastle–Ottawa Scale (NOS) was used to evaluate the quality of including non-controlled trials [12], and the retrospective studies were assessed by the JBI Critical Appraisal Checklist for Case Series [13].

### Statistical analysis

All statistical analyses and data manipulations were conducted using R v.4.1.0 (http://www.R-project.org/). Heterogeneity among studies was evaluated using chi-squared test and I^2^ statistics. p<0.05 indicated a statistically significant difference. If significant heterogeneity (p-value<0.05 or I^2^>50 %) existed, a random-effect model was performed. Otherwise, the fixed-effects model was used. Otherwise, heterogeneity was considered insignificant. Sensitivity analysis was performed to evaluate the stability of the research results. Publication bias was assessed using funnel plots. The source of bias was evaluated in subgroup analyses if there was a significant risk of bias.

### Compliance with ethical standards

This systematic review was conducted in accordance with the Preferred Reporting Items for Systematic Reviews and Meta-Analyses (PRISMA) reporting guidelines, and was registered with PROSPERO (CRD42024575313).

## Results

### Study selection

**Figure 1:**
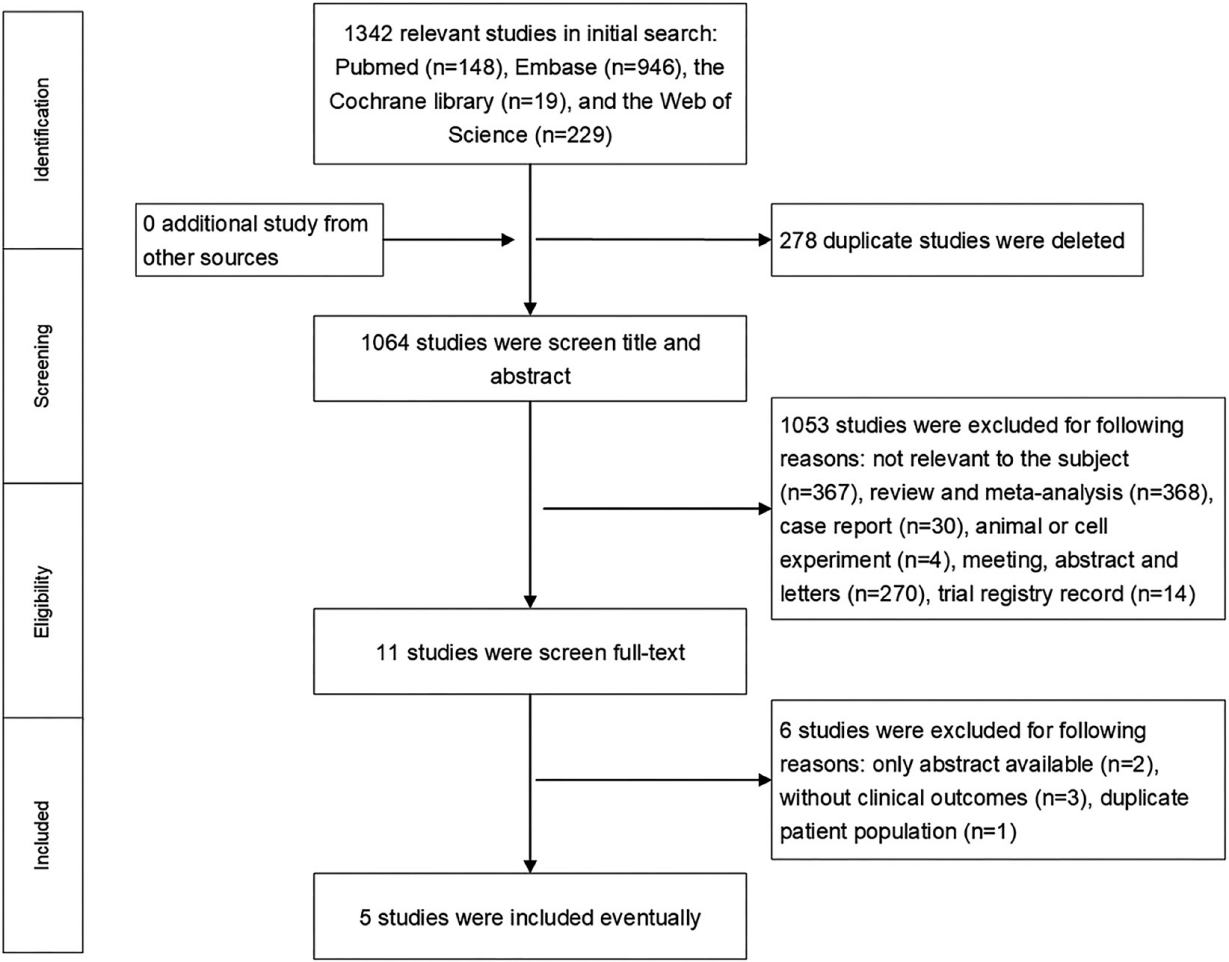
Flow diagram of meta-analysis for inclusion/exclusion of studies.

The initial search yielded a total of 1,342 published relevant studies from four databases (PubMed=148, Embase=946, Web of Science=229, and Cochrane Library=19). After deleting duplicate articles and screening, five studies with a total of 107 patients met the inclusion criteria and were included in this meta-analysis [14], [15], [16], [17], [18]. Of these patients, 65 had GCA and 42 had TAK. The Baricitinib was used in 4 studies [14], [16], [17], [18], Tofacitinib was used in 3 studies [15], [16], [17], and Upadacitinib was used only in one study [17]. The flowchart of the selection process is shown in Figure 1, and the details of each included study are described in Table 1.

**Table 1:** Characteristics of the studies included in the meta-analysis.

Study (year)	Country	Research type	Number of cases (male/female)	Age (years)	Median follow-up, months	JAK inhibitors received	Type of LVV	Previous biologic drugs	Outcomes
Koster et al. [14]	USA	Prospective	15 (4/11)	72.4 ± 7.2	11.3 ± 2.9	Baricitinib	GCA	Sirukumab (n=1)	Remission, AE, relapse, change in ESR, CRP, and GC dose
Wang et al. [15]	China	Prospective	32 (6/26)	30.9 ± 9.0	12	Tofacitinib	TAK	≥2 conventional immunosuppressants (n=32)	Remission, AE, relapse, change in ESR, CRP, GC dose, and vascular progression,
Eriksson et al. [16]	Sweden	Retrospective	15 (7/8)	70.2 ± 6.0	19.0 ± 10.5	Tofacitinib/Baricitinib	GCA	Tocilizumab (n=3), Infliximab (n=1)	Remission, AE, relapse, change in ESR, CRP, and GC dose
Loricera et al. [17]	Spain/USA	Retrospective	35 (5/30)	72.3 ± 8.0	11.4 ± 6.8	Baricitinib/Tofacitinib/Upadacitinib	GCA	Tocilizumab (n=26), Sarilumab (n=3), Abatacept (n=8), Adalimumab (n=2), Ustekinumab (n=2)	Remission, AE, relapse, change in ESR, CRP, and GC dose
Zhou et al. [18]	China	Prospective	10 (1/9)	29.7 ± 8.6	14.0 ± 9.5	Baricitinib	TAK	Secukinumab (n=3), TNF-α inhibitor (n=2), Tocilizumab (n=2), Tofacitinib (n=1)	Remission, AE, relapse, change in ESR, CRP, and GC dose

AE, adverse event; CRP, C reactive protein; ESR, erythrocyte sedimentation rate; GC, glucocorticoid; GCA, giant cell arteritis; LVV, large vessel vasculitis; TAK, Takayasu arteritis; TNF, tumor necrosis factor.

### Quality assessment

Four observational studies were assessed using the JBI Critical Appraisal Checklist for Case Series, which contains 10 items that assess the quality of case reports from the selection of cases. One non-randomized study was assessed using the NOS, which categorized studies into three dimensions, including population election, comparability, and outcome. The quality assessment details are shown in Supplementary Table S1.

### Remission

The present meta-analysis encompassed five studies, each with varying sample sizes of 14, 32, 11, 20, and 7 patients, all of whom were followed for 12 months and reported remission rates [14], [15], [16], [17], [18]. The Koster et al. study documented 13 patients achieving remission within the year, while Wang et al. reported 22 patients in complete remission and one in partial remission. Eriksson et al. utilized a broader definition of “therapeutical benefit,” encompassing patients with no suspicion of clinical activity, absence of new symptoms, and either decreasing or stable C reactive protein levels, resulting in 10 such cases. Loricera et al. reported 13 cases of complete remission and one of clinical remission. The study by Zhou et al. reported “complete response” in 3 patients and “partial response” in 1 patient. Given the heterogeneity in remission definitions across various studies, as outlined in Table 2, these varied terms of “remission” or “response” were harmonized and considered as remission for the purposes of the current study.

**Table 2:** The targets of definition of remission in included studies.

Authors (year)	Type of LVV	Target of remission	Definition
Koster et al. [14]	GCA	Clinical stability and remain in disease remission	Improvement in, or the absence of, ongoing signs or symptoms attributable to GCA as evidenced by reduction in symptoms and/or improvement in (or normalization of) inflammatory markers.
Wang et al. [15]	TAK	Complete remission	(i) no new symptoms or worsening of systemic symptoms; (ii) no new symptoms or worsening of vascular symptoms or signs; (iii) normal ESR (≤40 mm/h); (iv) GCs dose ≤15 mg/day (6 months) or ≤10 mg/day (12 months).
		Partial remission	Meeting criterion (ii) combined with any two of (i), (iii) and (iv)
Eriksson et al. [16]	GCA	Therapeutical benefit	Without suspicion of clinical activity and with the absence of new GCA symptoms (suspected to be related to GCA) and decreasing or stable CRP combined with decreasing daily prednisolone dose at 3- and 6-months post-initiation of JAK inhibitors.
Loricera et al. [17]	GCA	Clinical remission	The absence of signs and symptoms attributable to GCA regardless of the value of the ESR and CRP.
		Complete remission	The absence of signs and symptoms attributable to GCA and the normalization of the ESR and CRP values.
Zhou et al. [18]	TAK	Complete response	No evidence of active disease: (1) ESR<20 mm/h and CRP<10 mg/L, (2) no progression of vessel damage and (3) the dose of GC<15 mg/day prednisone (or equivalence).
		Partial response	(1) ESR<40 mm/h or decrease over 50 % compared with baseline, (2) CRP<20 mg/L or decrease over 50 % compared with baseline and (3) not fulfilling the other two criteria of complete response.

LVV, large vessel vasculitis; GCA, giant cell arteritis; TAK, Takayasu arteritis; ESR, erythrocyte sedimentation rate; GC, glucocorticoid; CRP, C reactive protein.

**Figure 2:**
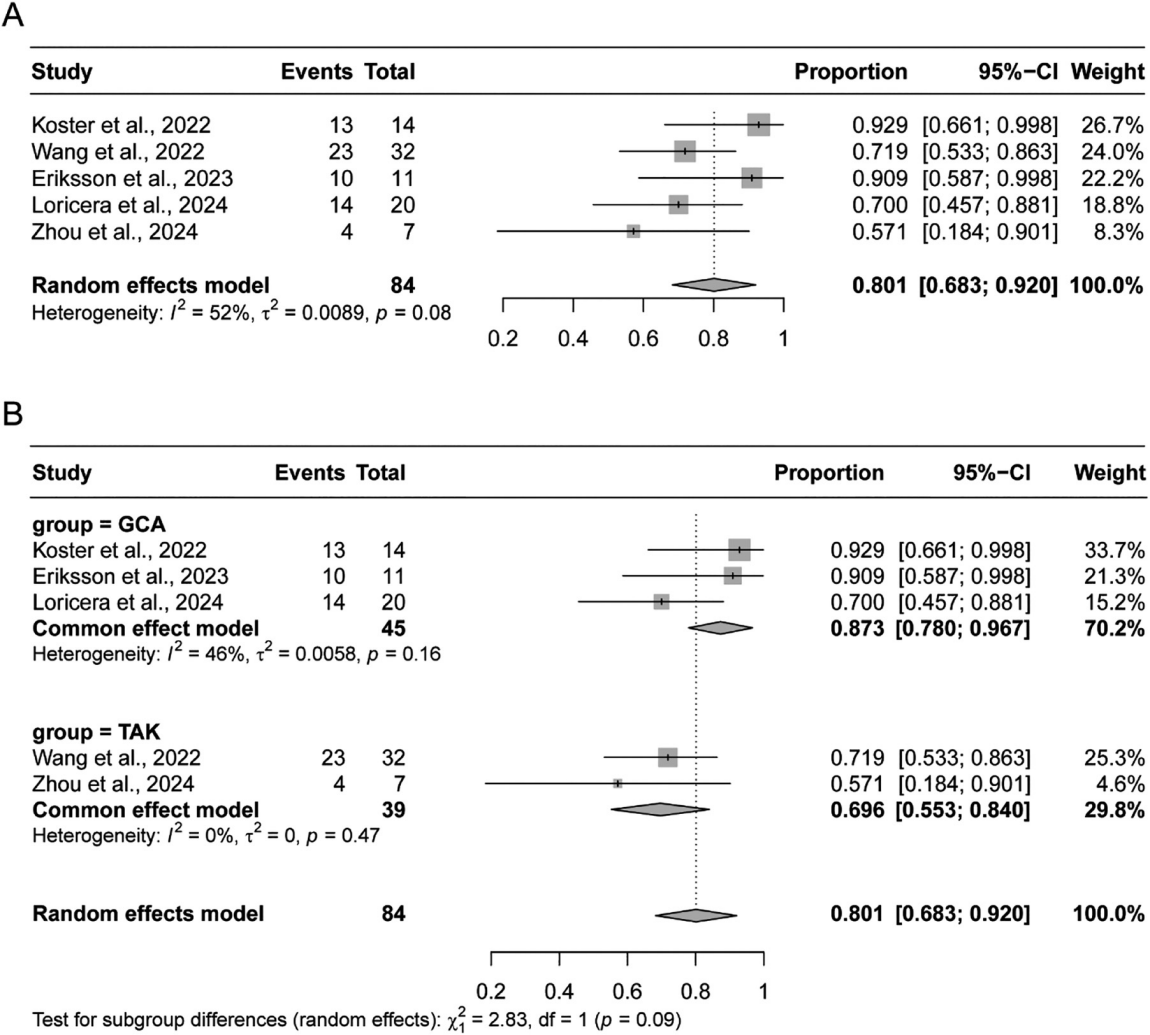
Pooled remission rates in patients with large vessel vasculitis (LVV). (A) Forest plot of the overall pooled remission rate across all 5 included studies. (B) Subgroup analysis of remission rates stratified by LVV type: giant cell arteritis (GCA, top) and Takayasu arteritis (TAK, bottom). GCA, giant cell arteritis; TAK, Takayasu arteritis.

The observed 12-month remission rates across the studies spanned from 70.0 % to 92.9 %, with a pooled remission rate of 80.1 % (95 % CI: 68.3–92.0 %), calculated using a random effects model due to moderate heterogeneity (I^2^=52 %, p=0.08) (Figure 2A). To further explore potential differences, subgroup analyses were conducted based on the type of LVV. Among the three studies focusing on GCA, remission rates were 70.0 %, 90.9 %, and 92.9 %, respectively, yielding a combined rate of 87.3 % (95 % CI: 78.0–96.7 %) under a fixed-effect model due to low heterogeneity (I^2^=46 %, p=0.16). In contrast, the two studies investigating TAK reported remission rates of 57.1% and 71.9 %, with a combined rate of 69.6 % (95 % CI: 55.3–92.0 %), also analyzed using a fixed effects model due to a lack of heterogeneity (I^2^=0 %, p=0.47) (Figure 2B).

### Relapse

**Figure 3:**
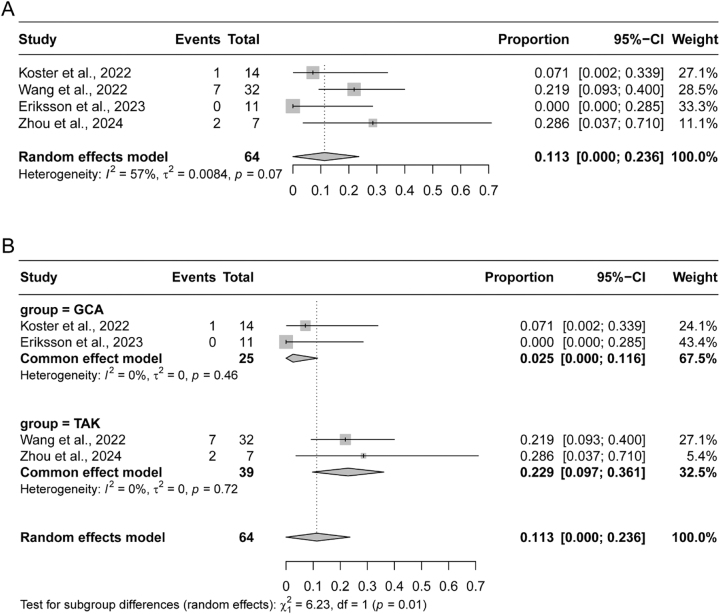
Pooled relapse rates in patients with large vessel vasculitis (LVV). (A) Forest plot of the overall pooled relapse rate across all 5 included studies. (B) Subgroup analysis of relapse rates stratified by LVV type: giant cell arteritis (GCA, top) and Takayasu arteritis (TAK, bottom). GCA, giant cell arteritis; TAK, Takayasu arteritis.

Of the five included studies, four reported relapse rates of LVV after 12 months of treatment with JAK inhibitors, ranging from 0 % to 28.6 % [14], [15], [16, 18]. Given the observed heterogeneity (I^2^=57 %, p=0.07), a random-effects model was employed to calculate a combined relapse rate of 11.3 % (95 % CI: 0–23.6 %) (Figure 3A). Subgroup analyses, performed separately for giant cell arteritis (GCA) and Takayasu arteritis (TAK), adopted fixed-effect models due to the homogeneity within each subgroup. In the GCA subgroup, comprising two studies, the pooled relapse rate was 2.5 % (95 % CI: 0–11.6 %), and the TAK subgroup, also consisting of two studies, exhibited a combined relapse rate of 22.9 % (95 % CI: 9.7–36.1 %) (Figure 3B).

### Adverse events

Across all five studies included in our meta-analysis, adverse events associated with JAK inhibitor therapy were reported, while one study provided cumulative adverse event data for the entire study duration instead of 12-month [17]. Koster et al. detailed a comprehensive list of adverse events, including infection not requiring antibiotics (n=8), infection requiring antibiotics (n=5), nausea (n=6), leg swelling (n=2), fatigue (n=2), diarrhea (n=1), abdominal pain (n=1), symptomatic herpes zoster (n=1), COVID-19 (n=2), and transient thrombocytopenia (n=1). Two of them led to JAK inhibitor discontinuation. Wang et al. observed herpes zoster infections (n=2) and a lipid level elevation (n=1), with viral infections prompting cessation of treatment. Eriksson et al. reported a single case of Aspergillus Fumigatus infection (n=1) at 12 months, with Loricera et al. reported the adverse events for the entire period of their study, one case developed urinary tract infection without requiring permanent JAK inhibitor discontinuation, and other adverse events led to drug discontinuation with significant elevation of liver enzymes (n=1), palpitations and dyspnea (n=1), disseminated herpes zoster (n=1), and glioblastoma multiforme (n=1). The study of Zhou et al. reported no severe adverse events occurred during the study period.

**Figure 4:**
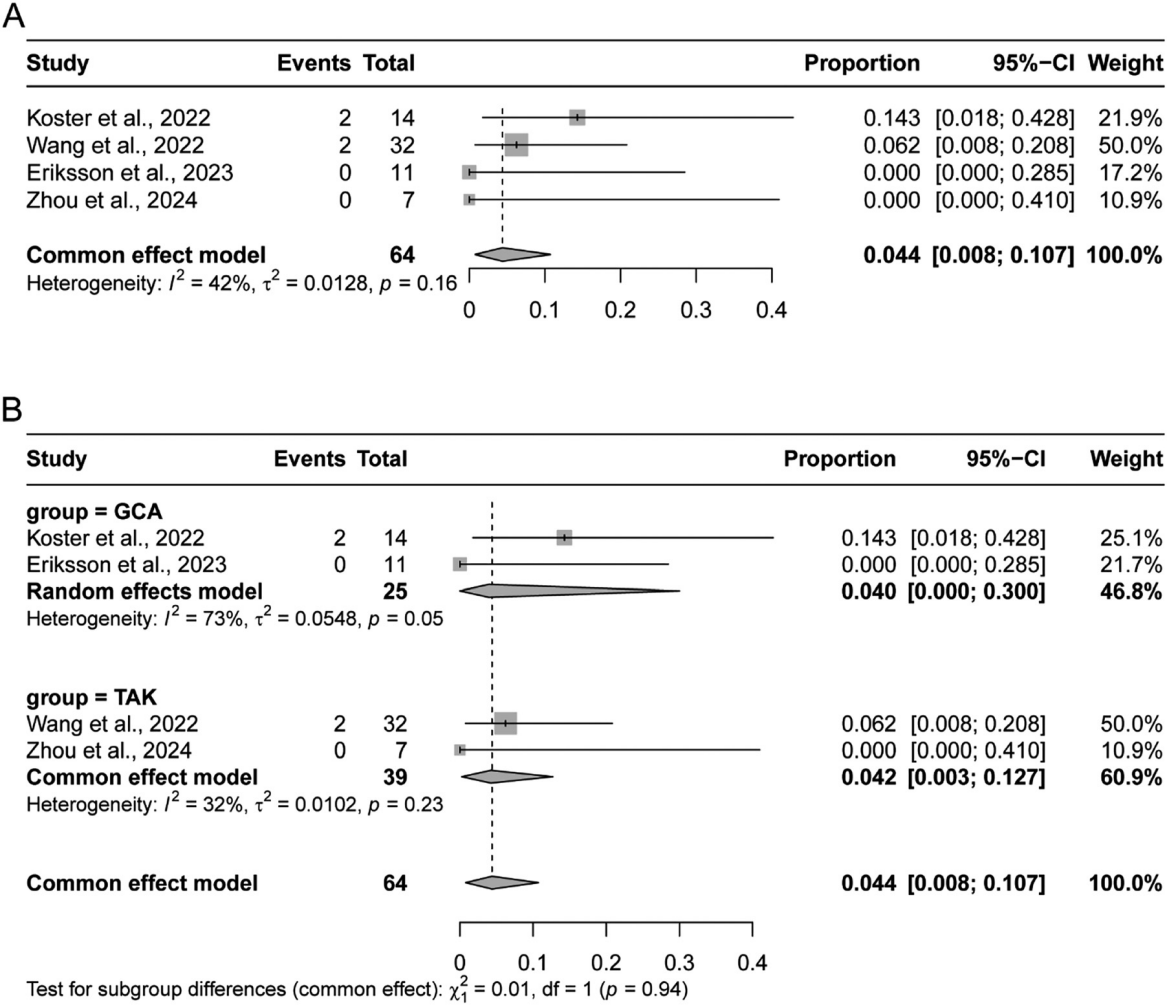
Pooled adverse event rates in patients with large vessel vasculitis (LVV). (A) Forest plot of the overall pooled adverse event rate across all 5 included studies. (B) Subgroup analysis of adverse event rates stratified by LVV type: giant cell arteritis (GCA, top) and Takayasu arteritis (TAK, bottom). GCA, giant cell arteritis; TAK, Takayasu arteritis.

Given the inconsistencies in adverse event definitions and reporting methodologies, we focused our analysis on adverse events requiring JAK inhibitor discontinuation at 12 months. Utilizing a fixed-effects model due to moderate heterogeneity (I^2^=42 %, p=0.16), we calculated a combined incidence of 4.4 % (95 % CI: 0.8–10.7 %) (Figure 4A). Subgroup analyses revealed distinct patterns: in the GCA subgroup, the two studies reported adverse event rates of 14.3 % and 0 %, yielding a combined rate of 4.0 % (95 % CI: 0–30 %) under a random-effects model due to high heterogeneity (I^2^=73 %, p=0.05). Conversely, the TAK subgroup showed a combined rate of 4.2 % (95 % CI: 0.3–12.7 %), calculated using a fixed-effects model owing to low heterogeneity (I^2^=32 %, p=0.23), with adverse event rates of 6.2 % and 0 % in the two studies (Figure 4B).

### Sensitivity and publication bias analysis

To evaluate the influence of individual studies on the consolidated outcomes, a sensitivity analysis was undertaken, involving the sequential exclusion of each study. The analysis findings revealed that none of the aggregated results, accompanied by 95 % CIs, underwent notable alterations due to the exclusion of any single study. This underscores the overall robustness and reliability of the conclusions drawn from this meta-analysis. The results of sensitivity analysis are shown in Supplementary Figures S1–3. Furthermore, the rate of remission, relapse, and adverse event were assessed by funnel plot, which showed no publication bias (Supplementary Figures S4–6).

## Discussion

This meta-analysis comprehensively reviewed the existing literature on the efficacy and safety of JAK inhibitors in the treatment of LVV. Our results showed that 80.1 % of LVV patients treated with JAK inhibitors for 12 months achieved remission, compared with an overall recurrence rate of 11.3 % and a rate of only 4.4 % of adverse events leading to discontinuation. The results of the study indicate that JAK inhibitors may be one of the promising drugs for treating LVV. Quality-related bias is minimal: High-quality studies (Wang et al. [15], NOS=8/9) contributed 30.8 % of total patients (32/107), and sensitivity analysis showed no major impact of low-quality studies on pooled results.

LVV is a complex autoimmune disease that includes GCA and TAK. Treatment strategies for LVV have changed dramatically over the past few decades. Currently, GC are still the cornerstone of treatment for LVV, but long-term use of GC is associated with significant side effects, with more than half of patients having at least one GC-related adverse event [5], which is associated with disease recurrence after stopping GC [19]. As the research background, GCA -related studies have found no significant benefit from methotrexate and tumor necrosis factor (TNF) -α inhibitors [20], [21], [22]. To date, only interleukin-6 inhibitors (tocilizumab) have shown safety and efficacy in reducing recurrence and reducing the need for GC [23]. Tocilizumab has been rapidly integrated into clinical practice and incorporated into recently updated consensus management guidelines [3]. Although there is a significant improvement compared to GC monotherapy, the attack rate in GCA patients treated with tocilizumab is still about 20 % [24]. In addition, clinical trial and observational data show that at 12 months of treatment with tocilizumab, more than one-third of patients have not achieved sustained clinical response [23], 25]. Studies on TAK have shown that classical synthetic disease-modifying antirheumatic drugs (csDMARDs) can be used to help reduce GCs gradually while maintaining the disease in remission [26]. Therefore, in clinical practice, conventional synthetic csDMARDs such as azathioprine, leflunomide, motecophenolate, and tacrolimus are the options for inducing and maintaining remission in TAK treatment. However, there are still many refractory patients who relapse even after receiving GCs in combination with conventional csDMARD. Biological DMARDs (bDMARDs) (tumor necrosis factor inhibitors, tocilizumab, or secukinumab) have been reported to be effective in the treatment of TAK [27], 28]. But some patients are resistant to these bDMARDs. Therefore, the treatment of LVV remains challenging.

JAK inhibitors have attracted much attention from rheumatologists because of their successful use in rheumatoid arthritis (RA) [29] and spinal arthritis [30], even when compared to biologic therapies, it has obvious advantages. Since the first trial of tofacitinib for the treatment of RA [31], JAK inhibitors have shown efficacy in a variety of autoimmune and autoinflammatory diseases [32] and have become an important weapon in the fight against inflammatory rheumatism. There are also numerous studies currently exploring their use in lupus [33] and small vasculitis [34]. JAK inhibitors are synthetic small molecule compounds that inhibit the JAK-STAT pathway. They inhibit the activation of a variety of immune cells (effector T cells, macrophages, NK cells) and reduce the release of type I pro-inflammatory cytokines [35]. *In vitro* studies have shown that JAK inhibitors can inhibit the activation of the outer aortic membrane fibroblasts and reduce vasculitis [36]. In inflammatory lesions of LVV, Th1 and Th17 macrophages and CD4 + cells are the main immune cell effectors present in LVV lesions, producing cytokines via the JAK-STAT pathway, which in turn amplify the inflammatory response in a JAK-STAT dependent manner, thus creating a vicious cycle [37], 38]. Basic studies have shown that JAK-STAT signaling pathway is abnormally activated in immunodeficient humanized mouse models implanted in human arteries, and *in vitro* treatment with JAK inhibitors can effectively inhibit T cells with arterial wall lesions and reduce polarization to Th1 and Th17 cells, thereby reducing cytokine activation. It also inhibits macrophage infiltration and the production of growth factors, thereby reducing new angiogenesis and intimal hyperplasia [7], 10]. Therefore, we can control LVV inflammatory progression by targeting the JAK-STAT pathway. Tofacitinib, currently used as an JAK1/JAK3 inhibitor, has been found to induce TAK remission and prevent recurrence in observational studies [39]. Compared with Tofacitinib, Baricitinib has stronger inhibitory effect on JAK2. One study showed that IL-12 and IL-23 are central to TAK pathogenesis, and these cytokines activate downstream JAK2 [40]. Therefore, targeting JAK2 may achieve the same or better efficacy in patients with refractory TAK. Baricitinib is an oral and reversible JAK1/JAK2 inhibitor that has been approved for the treatment of RA [41]. Studies have also shown that baricitinib has demonstrated its efficacy and safety in the treatment of GCA [14].

The results of this study showed that JAK inhibitors are a relatively safe drug and have the potential to further expand their application. Given that the number of studies included in the subgroup analysis is very small, we need to analyze these results with caution. This provides us with a direction for research and treatment, but a large amount of new large-sample data is still needed to verify the above results.

It is worth noting that the five included studies had different definitions of remission. To obtain the most applicable and reliable results, we treated all “complete remission”, “partial remission”, and “therapeutic benefit” in the studies as remission outcomes. This combined remission is more inclined to clinical remission as they all have at least an improvement in clinical symptoms. However, it should be pointed out that this combination may overestimate the true remission rate because we have included as many remission patients as possible. This also leads to the conclusion that the evidence in this study is preliminary and hypothesis-generating rather than conclusive.

On the other hand, the small number of research in this study also limits statistical power and generalizability. The TAK subgroup has limited generalizability, primarily due to its small sample size (n=42 across 2 studies) – with Zhou et al. [18] including only 7 patients – and the fact that both TAK studies (Wang et al. [15]; Zhou et al. [18]) focused on refractory cases; for instance, 3 out of 7 patients in Zhou et al. [18] were secukinumab-refractory, which may lead to overestimated relapse rates (22.9 %) and underestimated efficacy in treatment-naive patients. Furthermore, JAK inhibitor’s efficacy in specific TAK subgroups, such as pediatric TAK or TAK with aortic stenosis, remains unknown due to a lack of relevant data. Future studies should not only include mechanistic biomarkers – such as STAT3 phosphorylation in peripheral blood mononuclear cells and serum IL-6/IFN-γ – to predict JAK inhibitor response, but also incorporate patient-reported outcomes (e.g., fatigue scores, quality of life measured via the SF-36) to capture patient-centered benefits, which are currently unreported in the included studies; additionally, these studies will further require multi-center randomized controlled trials, standardized outcomes, longer follow-up periods, and head-to-head comparisons with standard therapies to clarify the role of JAK inhibitors in vasculitis.

JAK inhibitors are emerging as a promising option for LVV treatment, but they are not the only treatment options being explored. Other drugs, such as TNF-α inhibitors and IL-6 inhibitors, however, direct comparisons of JAK inhibitors with these alternative drugs are rare and further research is needed to determine the best treatment for individual patients.

It must be acknowledged that there are several limitations in this meta-analysis. First, the number of eligible studies was small and the total sample size was small, which may have limited the generality of the findings. In addition, differences in mitigation and adverse event definitions between studies pose challenges for data interpretation and synthesis. Due to the lack of direct comparisons with other treatment options, no definitive conclusions can be drawn about the best treatment strategy. Several studies only report 12-month outcomes, lacking data on long-term efficacy (e.g., 24-month remission rate) and safety (e.g., long-term infection risk, vascular damage progression). JAK inhibitor’s role in organ protection (e.g., preventing aortic aneurysm in GCA) remains unaddressed, as no study reported imaging-based damage endpoints. Future studies should include ≥24-month follow-up and assess vascular damage via CTA/MRI. And it is possible that studies from non-English literature or small databases were not included, which may have an impact on the synthesis and potential biases. Finally, funnel plot-based publication bias assessment is unreliable due to only 5 included studies; future meta-analyses with ≥10 studies are needed to validate publication bias.

## Conclusions

In summary, this meta-analysis provides evidence of the efficacy and safety of JAK inhibitors in the treatment of LVV. JAK inhibitors have shown a high response rate and a low recurrence rate, along with a low incidence of adverse events. Further research, including randomized controlled trials and direct comparisons with other treatment options, is needed to fully elucidate the role of JAK inhibitors in the treatment of LVV.

## Supplementary Material

Supplementary Material
