# Supplementary material for: Janus Kinase inhibitors in the treatment of large vessel vasculitis: a systematic review and meta-analysis
Source: Open Med (Wars). 2026 Mar 6;21(1):20261387. doi: 10.1515/med-2026-1387 (PMC12962734; doi:10.1515/med-2026-1387)

**Supplementary Table S1. Quality assessment of the studies included in the meta-analysis.**

| Study | Quality assessment with JBI Critical Appraisal Checklist | | | | | | | | | |
| --- | --- | --- | --- | --- | --- | --- | --- | --- | --- | --- |
|  | Q1 | Q2 | Q3 | Q4 | Q5 | Q6 | Q7 | Q8 | Q9 | Q10 |
| Koster et al., 2022 | YES | YES | YES | UNCLEAR | UNCLEAR | NO | YES | YES | NO | YES |
| Eriksson et al., 2023 | YES | YES | YES | UNCLEAR | UNCLEAR | NO | YES | YES | NO | YES |
| Loricera et al., 2024 | YES | YES | YES | UNCLEAR | UNCLEAR | NO | YES | YES | NO | YES |
| Zhou et al., 2024 | YES | YES | YES | YES | YES | NO | YES | YES | NO | YES |
|  | Quality assessment with NOS (Newcastle–Ottawa Scale) | | | | | | | | | |
|  | QI | QII | QIII | QIV | QV | QVI | QVII | QVIII |  |  |
| Wang et al., 2022 | 1 | 1 | 1 | 1 | 2 | 1 | 1 | 1 |  |  |

Numbers Q1-10: Q1, Were there clear criteria for inclusion in the case series? Q2, Were there clear criteria for inclusion in the case series? Q3, Were valid methods used for identification of the condition for all participants included in the case series? Q4, Did the case series have consecutive inclusion of participants? Q5, Did the case series have complete inclusion of participants? Q6, Was there clear reporting of the demographics of the participants in the study? Q7, Was there clear reporting of clinical information of the participants? Q8, Were the outcomes or follow up results of cases clearly reported? Q9, Was there clear reporting of the presenting site(s)/clinic(s) demographic information? Q10, Was statistical analysis appropriate?

Numbers Q I-VIII: QI, Representativeness of the exposed cohort; QII, Selection of the non-exposed cohort; QIII, Ascertainment of exposure; QIV, Demonstration that outcome of interest was not present at start of study; QV, Comparability of cohorts on the basis of the design or analysis; QVI, Assessment of outcome; QVII, Was follow-up long enough for outcomes to occur; QVIII, Adequacy of follow up of cohorts.

Supplementary Fig. S1. Sensitivity analysis of remission.


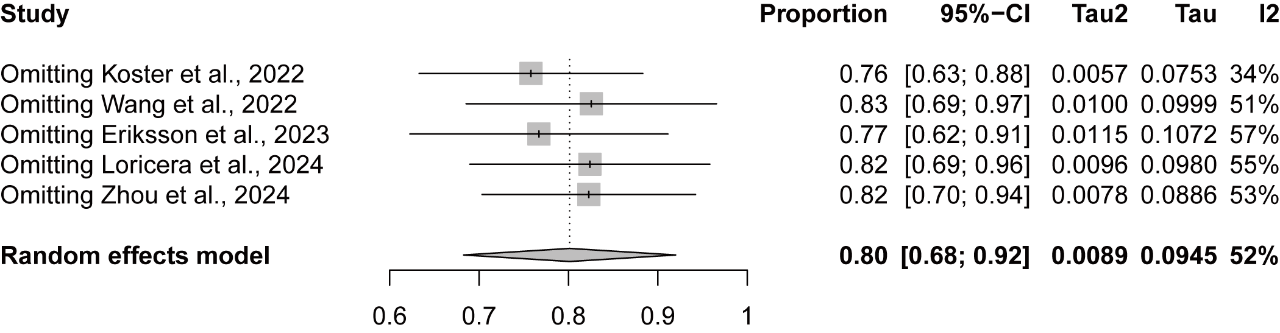


Supplementary Fig. S2. Sensitivity analysis of relapse.


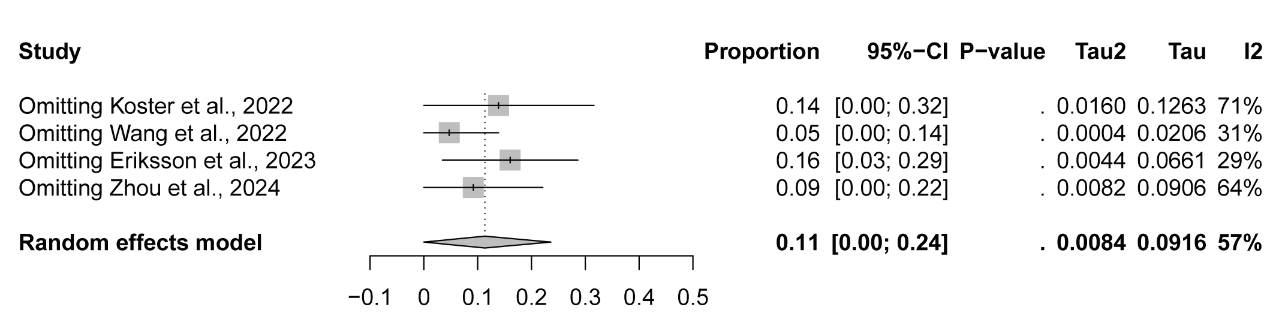


Supplementary Fig. S3. Sensitivity analysis of adverse events.


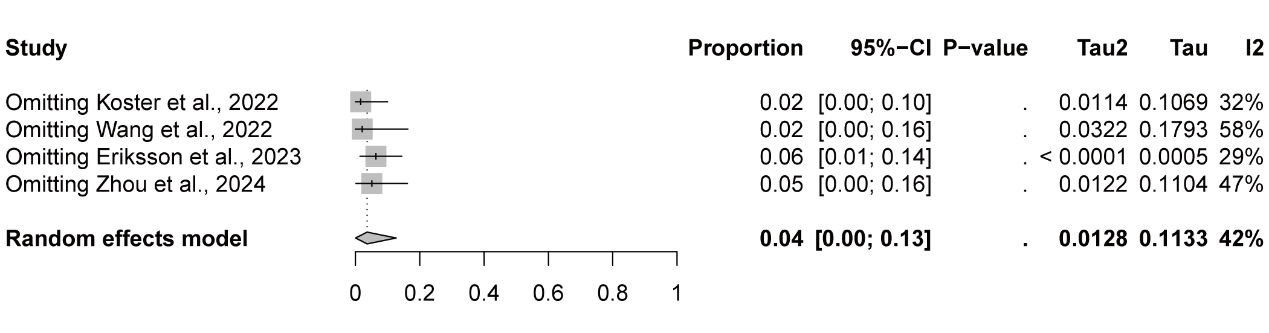


Supplementary Fig. S4. Funnel plots of remission.


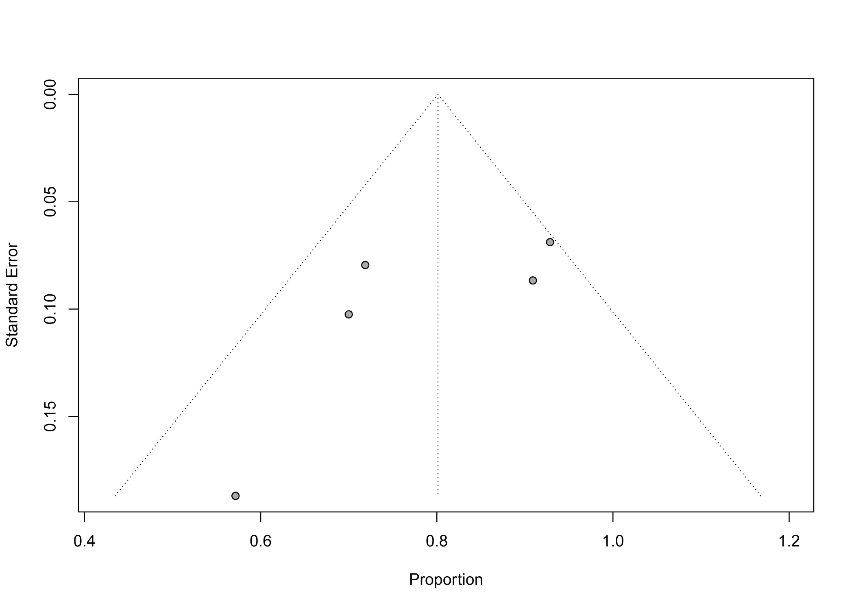


Supplementary Fig. S5. Funnel plots of relapse.


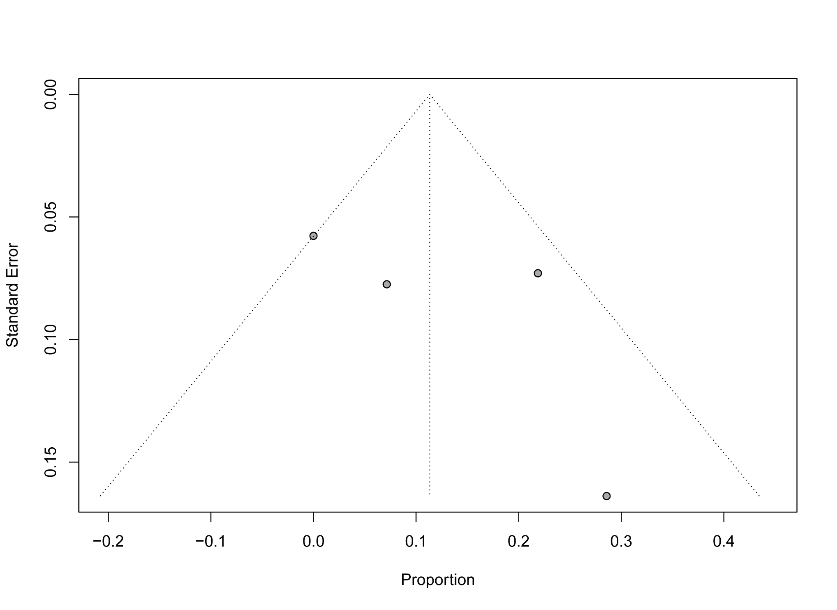


Supplementary Fig. S6. Funnel plots of adverse events.


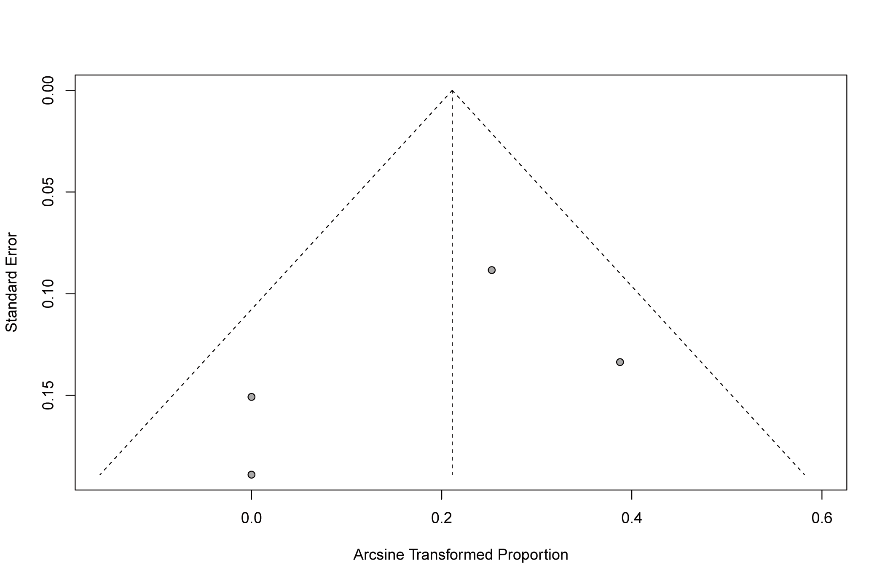

Supplement: Supplementary file 1 — Supplementary Material [file j_med-2026-1387_suppl_001.docx]
